# Erector Spinae Plane Block for Percutaneous Transhepatic Biliary Drainage: A Comparative Analysis

**DOI:** 10.1007/s00270-025-04108-5

**Published:** 2025-06-24

**Authors:** Çağrı Erdim, Mehmet Hamza Türkcanoğlu, Rabia Deniz, Hamit Özgül, Zöhre Okur, Tevfik Güzelbey, Mustafa Fatih Arslan, Özgür Kılıçkesmez

**Affiliations:** 1 Department of Radiology, University of Health Sciences Başakşehir Çam and Sakura City Hospital, Başakşehir Olimpiyat Bulvarı Yolu, 34480 Istanbul, Turkey; 2 Department of Radiology, University of Health Sciences Gülhane Training and Research Hospital, Ankara, Turkey; 3 Department of Rheumatology, University of Health Sciences Başakşehir Çam and Sakura City Hospital, Istanbul, Turkey; 4 Department of Medical Biology and Genetics, Marmara University, Istanbul, Turkey; 5 Department of Radiology, Medicana Ataköy Hospital, Istanbul, Turkey

**Keywords:** Percutaneous transhepatic biliary drainage, Erector spinae plane block, Procedural analgesia with fentanyl, Numeric Rating Scale, Analgesia, Procedural pain

## Abstract

**Purpose:**

Percutaneous transhepatic biliary drainage (PTBD) is associated with significant procedural pain, typically managed with opioid-based sedation, which carries risks such as respiratory depression, nausea, and hemodynamic instability. The erector spinae plane block (ESPB) has emerged as an opioid-sparing alternative for perioperative pain management. This study aimed to evaluate the analgesic efficacy of ESPB compared to procedural analgesia with fentanyl (PAF) in PTBD patients.

**Methods:**

Patients who underwent PTBD with ESPB or PAF were assessed using the Numeric Rating Scale (NRS) at five time points: pre-procedure, intra-procedure, and 1, 6, and 12 h post-procedure. Opioid consumption and procedure-related complications were also recorded.

**Results:**

101 patients who underwent PTBD with either pre-procedural ESPB (n = 41) or PAF (n = 60) were included. The ESPB group demonstrated significantly lower median pain scores at 1 h (3 vs. 6, *p* < 0.001), 6 h (2 vs. 4, *p* < 0.001), and 12 h (1 vs. 2, *p* < 0.001) post-procedure compared to the PAF group. Although intra-procedural pain scores were comparable between the two groups, patients in the ESPB group experienced a more rapid decline in post-procedural pain, returning to near-baseline levels at 1 h, whereas pain in the PAF group remained elevated (*p* < 0.001). No patients in the ESPB group required additional opioid analgesia post-procedure, whereas tramadol was administered in the PAF group as needed for breakthrough pain (NRS ≥ 6).

**Conclusion:**

ESPB provides effective analgesia for PTBD, minimizing opioid use while enhancing patient comfort and procedural success.

**Graphical Abstract:**

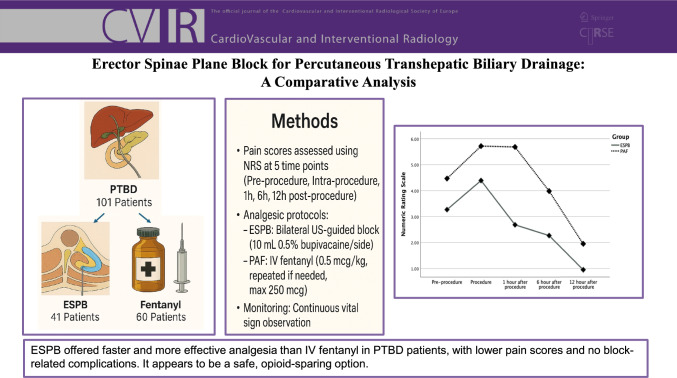

## Introduction

Percutaneous transhepatic biliary drainage (PTBD) is a widely used interventional radiology procedure for the management of both malignant and benign biliary obstructions. It plays a crucial role in relieving biliary stasis, reducing the risk of cholangitis, and improving liver function, particularly in patients with advanced malignancies [1]. Despite its clinical benefits, PTBD is associated with significant procedural pain, which can negatively impact patient comfort and procedural success [2, 3]. Traditionally, opioids such as fentanyl are administered for sedation and analgesia; however, opioid-related side effects, including respiratory depression, nausea, and hemodynamic instability, necessitate the search for alternative analgesic techniques [4].

Erector spinae plane block (ESPB), first described by Forero et al. in 2016, has emerged as a promising ultrasound-guided regional anesthesia technique that provides effective analgesia by targeting the dorsal and ventral rami of spinal nerves [5]. Initially developed for thoracic pain management, ESPB has gained widespread attention as an opioid-sparing alternative in various interventional and surgical procedures, including abdominal and hepatobiliary interventions [5–7]. The ability of ESPB to maintain hemodynamic stability and provide prolonged analgesia has been demonstrated in surgical patients [8], with comparable efficacy reported in similar percutaneous liver-directed procedures such as percutaneous cholecystostomy [9].

While the efficacy of ESPB in surgical settings has been well-documented, its role in percutaneous procedures such as PTBD remains largely unexplored. Given the high pain burden associated with PTBD and the limitations of opioid-based sedation, investigating whether ESPB can enhance analgesic outcomes, reduce opioid consumption, and minimize procedure-related complications is of clinical importance. This study aims to compare pre-procedural ESPB with intra-procedural fentanyl administration for pain management during PTBD, evaluating its impact on pain scores, opioid requirements, and overall patient experience.

## Materials and Methods

### Study Design

This retrospective, single-center, two-arm, observational study was conducted to compare the efficacy of ESPB and procedural analgesia (PA) with fentanyl (PAF) in pain management during PTBD. Ethical approval (approval number: 2024-316) for the study was obtained from the institutional review board. Informed consent for the procedure and data publication was obtained from all patients.

### Patient Selection

PTBD was routinely performed under PAF. However, ESPB was applied in patients who either declined sedation or were deemed at risk for mild-to-moderate sedation based on their vital signs and clinical status (including elderly patients with hypovolemia, liver failure, or renal failure). As this was a retrospective study, randomization was not performed, and group allocation was based on clinical judgment and patient-specific risk profiles. Patient data from January 2023 to November 2024 were collected.

#### Inclusion Criteria

- Patients requiring PTBD due to biliary obstruction or bile duct injury,
- ECOG performance status of 0–2,
- No contraindications to local anesthesia or PA,
- Patients without history of chronic opioid use or pre-existing neuropathic pain.

#### Exclusion Criteria

- Patients who underwent internal–external drainage catheter placement (excluded to ensure group comparability, as these catheters traverse longer liver segments and may cause higher pain levels),
- Patients who underwent bilateral drainage catheter placement,
- Patients with missing pre-procedure laboratory values,
- Patients unable to provide reliable pain scores,
- Patients who developed allergic reactions or intolerance to ESPB medications,
- Patients with severe coagulopathy (International Normalized Ratio (INR) > 2, platelet count < 50,000/mm^3^),
- Patients with prior hepatobiliary surgeries that may affect pain perception,
- Patients with sepsis or hemodynamic instability.

### Data Collection and Outcome Measures

Demographics, procedure details including laterality, puncture site, and common bile duct diameter, and laboratory parameters were collected. Pain scores were assessed using the Numeric Rating Scale (NRS) at five time points: pre-procedure (60 min before PTBD, prior to ESPB administration), during the procedure, and post-procedure (1 h, 6 h, and 12 h after PTBD), based on the pain reported by the patient. The NRS score ranged from 0 to 10, with 0 indicating no pain and 10 representing the worst imaginable pain. Complications during procedure and follow-up were also recorded.

In our department, pain assessments using the Numeric Rating Scale (NRS) are routinely performed for all patients undergoing interventional procedures, including PTBD, as part of standard clinical care. Pain scores are systematically recorded at predefined time points: pre-procedure, intra-procedure, and at 1, 6, and 12 h post-procedure.

Although this study was retrospective in design, the pain scores were prospectively and systematically collected during routine clinical practice. Importantly, no retrospective patient interviews or recall-based assessments were conducted; all data were retrieved directly from contemporaneously documented medical records. The retrospective nature of this study pertains solely to the subsequent retrieval and analysis of these routinely collected data, without prospective randomization or group allocation.

Pain management protocols included administration of additional opioids (tramadol) if NRS scores reached 6 or higher. This threshold was applied consistently during postoperative care.

### Procedural Techniques

#### Erector Spinae Plane Block (ESPB)

**Fig. 1:**
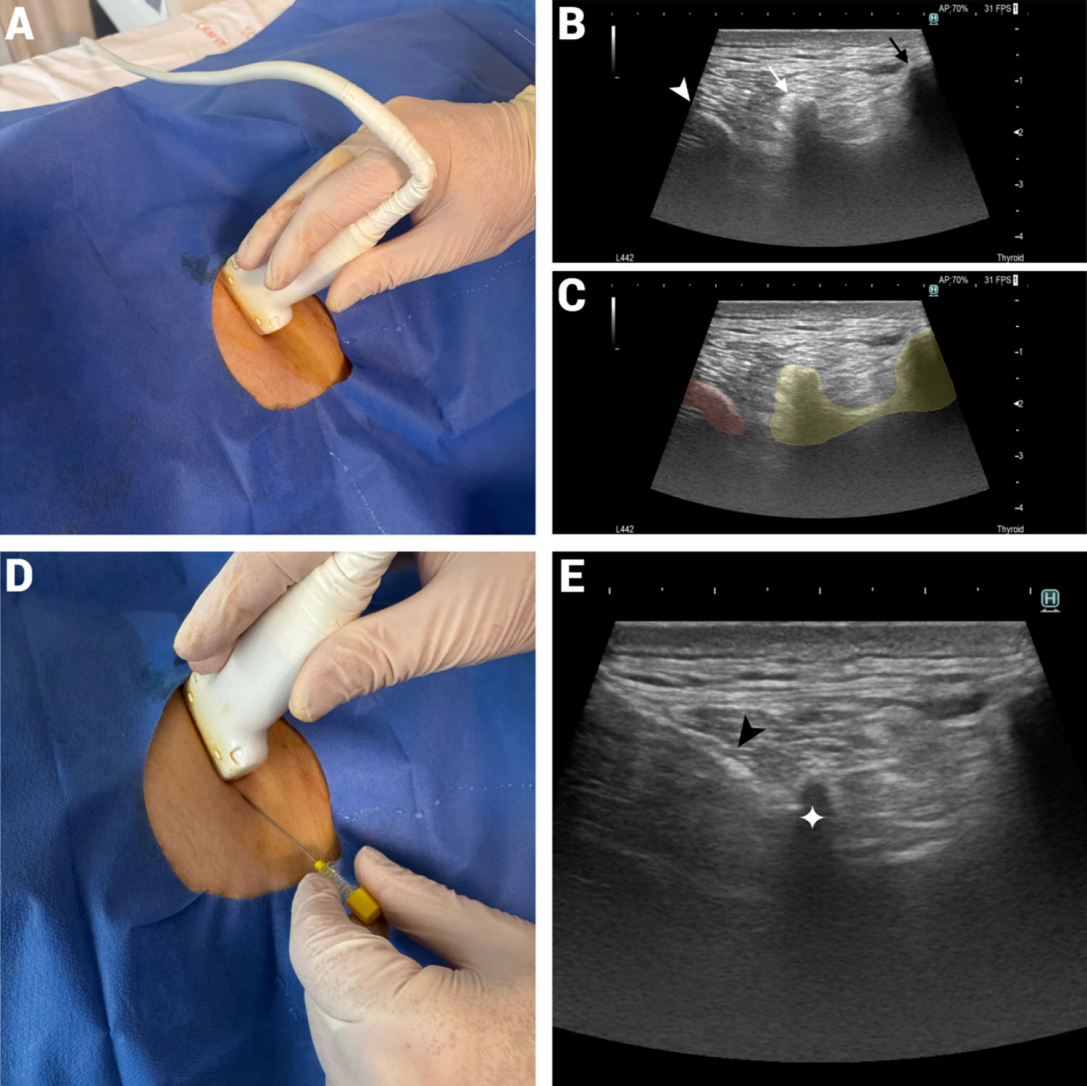
Ultrasound-Guided Erector Spinae Plane Block for Percutaneous Transhepatic Biliary Drainage (PTBD). **A** Ultrasound probe positioning at the T8 transverse process level before needle insertion **B** Ultrasound image showing key anatomical landmarks: the white arrowhead indicates the costa, the white arrow points to the transverse process, and the black arrow marks the spinous process **C** Annotated ultrasound image highlighting the costa in red and the posterior elements of the T8 vertebra in yellow **D** Needle insertion under ultrasound guidance for local anesthetic administration **E** Final ultrasound confirmation showing the correct placement of the needle (black arrowhead) within the fascial plane, with the needle tip (white star) touching the transverse process

ESPB was performed with the patient in the prone position using the Arietta 65 (Hitachi, Fujifilm, Tokyo, Japan) ultrasound system. A 5–18 MHz linear probe was used for thin patients, while a 1–5 MHz convex probe was preferred for obese patients to ensure clear visualization and precise needle placement (Fig. 1). The block was administered 60 min before PTBD. In all patients, the targeted insertion route via T8 transverse process was identified, and a 22G spinal needle was advanced into the interfascial plane between the erector spinae muscle and the transverse process. Hydrodissection was performed with 0.5 mL of saline in the right interfascial plane, and cranio-caudal spread was observed under ultrasound. After confirming the absence of vascular injury or unintended entry, 10 mL of 0.5% bupivacaine was injected. The same procedure was then repeated on the left side, and the spread of the local anesthetic was confirmed via ultrasound to ensure proper diffusion in the targeted fascial plane.

#### Procedural Analgesia with Fentanyl

For patients undergoing PAF, fentanyl was administered intravenously at an initial dose of 0.5 mcg/kg before local anesthesia. Additional fentanyl doses (0.5 mcg/kg increments) were given if the patient’s pain score exceeded 4 on the NRS, with a minimum interval of 5 min between doses. The maximum total dose was limited to 250 mcg. All patients were monitored continuously for oxygen saturation, respiratory rate, and hemodynamic stability throughout the procedure.

#### Percutaneous Transhepatic Biliary Drainage Procedure

PTBD was performed by a 6 years of experienced interventional radiologist using ultrasound and fluoroscopic guidance.Prophylactic antibiotics (1 g ceftriaxone) were administered intravenously one hour before the procedure. Following proper sterilization, local anesthesia with 10 mL of 1% lidocaine was applied to the puncture site.

A subxiphoid or subcostal approach was utilized for left lobe access, while an intercostal approach was preferred for right lobe access. Under ultrasound guidance, a 21G Chiba needle was used to puncture the bile duct, and diluted iodine contrast medium (Ultravist 300, Bayer AG) was injected to delineate the biliary anatomy. After successful biliary access, a 0.018-inch nitinol guidewire was advanced through the needle, allowing for the introduction of a 6F sheath. Subsequently, a 0.035-inch guidewire was passed through the sheath, which was then removed. Following guidewire placement, an external biliary drainage catheter was advanced over the wire and positioned according to the patient’s pathology. Proper positioning was confirmed through fluoroscopic imaging, and the catheter was secured to the skin. Antibiotic therapy was continued for 24 h post-procedure to minimize infection risk. Patients were assessed for breakthrough pain management within the first 12 h post-procedure.

### Statistical Analysis

Since the data distribution was heterogeneous, except for age, continuous variables are presented as median values with ranges, and categorical variables are presented as percentages. Differences in continuous variables between independent groups were analyzed using the Mann–Whitney U test with Yate’s correction, while differences in categorical data frequencies were analyzed using the χ^2^ test. Differences between two dependent measurements were evaluated using Wilcoxon’s test and Friedman’s test. Variance in repeated measurements during follow-up visits was assessed using Mauchly’s test for sphericity, with Greenhouse–Geisser correction applied when necessary. Group differences were analyzed using univariate MANOVA, applying Pillai’s trace test to minimize errors caused by heterogeneity and small sample size. A *p*-value of < 0.05 was considered statistically significant. Statistical analyses were performed using SPSS Statistics for Windows, version 30.0 (SPSS Inc., Chicago, IL, USA).

## Results

### Demographic and Clinical Characteristics

A total of 101 patients were enrolled in the study, including 41 patients in the ESPB group and 60 in the PAF group. Baseline characteristics and laboratory parameters are summarized in Table 1. The ESPB and PAF groups were comparable in terms of age, sex distribution, and most laboratory values. However, common bile duct (CBD) diameter was significantly smaller in the ESPB group than in the PAF group (10 mm vs. 13 mm, *p* = 0.007), and indirect bilirubin levels were lower in the ESPB group (0.90 mg/dL vs. 1.48 mg/dL, *p* = 0.019). Other laboratory parameters, including white blood cell count, hemoglobin, hematocrit, platelet count, and coagulation markers, did not show statistically significant differences between the groups (*p* > 0.05 for all).

**Table 1:** Patient characteristics and laboratory parameters before procedure

Parameters	ESPB group (n = 41)	PAF group (n = 60)	*p* value
Female/Male (n,%)	18/23 (43.9/56.1)	25/35 (41.7/58.3)	0.492
Age, year (median, range)	67 (40–89)	66.5 (39–94)	0.599
Choledocus diameter, mm (median-range)	10 (4–22)	13 (5–19)	**0.007**
Laterality, right/left (n, %)	15/26 (36.6/63.4)	16/44 (26.7/73.3)	0.200
Procedure site (n, %)			
Subcostal	32 (78.0)	47 (78.3)	0.580
Intercostal	9 (22.0)	13 (21.7)	
Primary etiology of obstruction (n, %)			
Benign liver pathology	8 (19.5)	16 (26.7)	0.459
Primary or metastatic liver cancer	16 (39.0)	26 (43.3)	
Pancreatic carcinoma	17 (41.5)	18 (30.0)	
WBC 10^6^/L (median, range)	8670 (1800–21260)	8150 (3390–21830)	0.793
Hemoglobin g/dL (median, range)	10.9 (7.3–13.4)	10.7 (7.3–15.3)	0.691
Hematocrit % (median, range)	32.5 (21.8–39.1)	31.9 (21.7–44.5)	0.898
Platelets 10^9^/L (median, range)	293 (107–704)	256.5 (54–516)	0.901
Total bilirubin, mg/dL (median, range)	10.38 (0.39–32.09)	11.58 (0.28–28.73	0.422
Direct bilirubin mg/dL (median, range)	9.55 (0.25–30.97)	9.83 (0.20–27.86)	0.625
Indirect bilirubin mg/dL (median, range)	0.90 (0.09–3.7)	1.48 (0.04–5.03)	**0.019**
ALT, U/L (median, range)	90 (14–466)	74.5 (2–469)	0.562
AST, U/L (median, range)	94 (10–385)	74.5 (19–432)	0.319
ALP, U/L (median, range)	512.5 (51–2115)	374.5 (121–1747)	0.132
GGT, U/L (median, range)	414.5 (31–1195)	337 (22–1829)	0.099
Amylase, U/L (median, range)	60 (16–681)	56 (11–676)	0.642
PT second (median, range)	10.4 (7.87–15.3)	10.9 (1.10–29.5)	0.953
INR (median, range)	1.1 (0.8–1.4)	1.2 (0.9–1.98)	0.560
APTT second (median, range)	32.4 (25.9–49.6)	33.9 (24.4–53.2)	0.252

Bold values indicate the statistically significant differences between groups

The primary etiologies of biliary obstruction in both groups are presented in Table 1. The most common cause was pancreatic carcinoma, accounting for 41.5% of cases in the ESPB group and 30.0% in the PAF group. Primary or metastatic liver cancer was the second most frequent etiology, observed in 39.0% of ESPB patients and 43.3% of PAF patients. Benign liver pathology, including choledocholithiasis and benign strictures, was less common, comprising 19.5% of cases in the ESPB group and 26.7% in the PAF group. There were no statistically significant differences between the groups in terms of the distribution of etiologies (*p* = 0.459), indicating a comparable underlying disease spectrum between the two study arms.

### Pain Scores

**Fig. 2:**
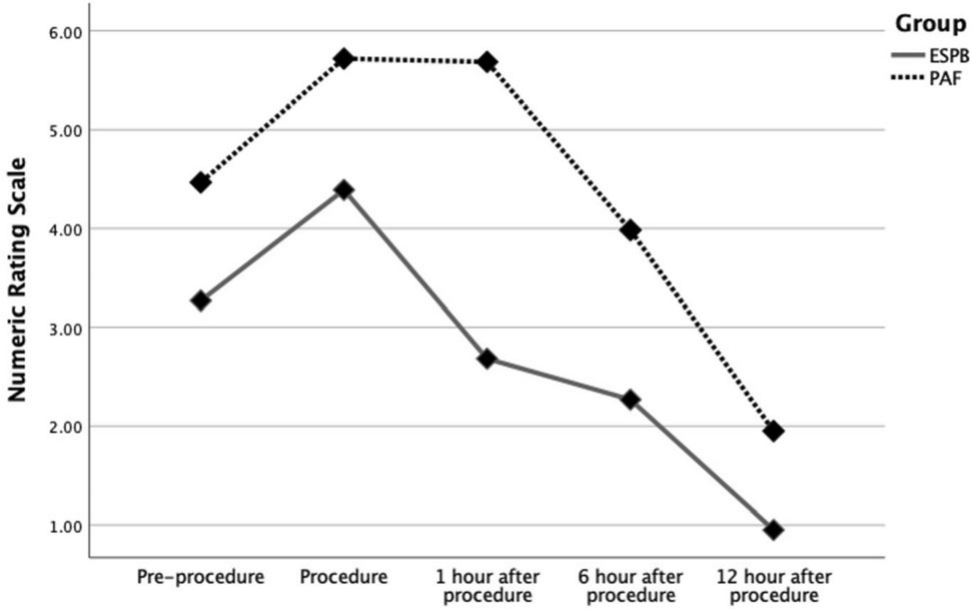
Median Numeric Rating Scale (NRS) scores at measurement point for the erector spinae plane block (ESPB) or procedural analgesia with fentanyl (PAF) groups

The median NRS scores at each measurement point are summarized in Fig. 2. Pre-procedural pain scores were slightly lower in the ESPB group compared to the PAF group [3 (range: 0–8) vs. 4 (range: 1–8); *p* > 0.05, inter-group]. During the procedure, both groups experienced an expected increase in pain scores [ESPB: 5 (0–9); PAF: 6 (2–9); *p* = 0.496, inter-group].

At 1 h post-procedure, the ESPB group showed a significant reduction in pain scores compared to baseline [pre-procedure: 3 (0–8) vs. 1st hour: 3 (0–5); *p* < 0.001, intra-group], while the PAF group did not demonstrate any significant decrease [pre-procedure: 4 (1–8) vs. 1st hour: 6 (2–9); *p* = 0.920, intra-group]. Between groups, pain scores at the 1st hour were significantly lower in the ESPB group [*p* < 0.001, inter-group].

At 6 h post-procedure, pain levels continued to decrease in both groups, with a more pronounced reduction in the ESPB group [2 (0–5) vs. 4 (0–7); *p* < 0.001, inter-group]. At 12 h, the ESPB group reported near-complete pain relief [1 (0–3)], while the PAF group still experienced higher residual pain [2 (0–4); *p* < 0.001, inter-group].

**Fig. 3:**
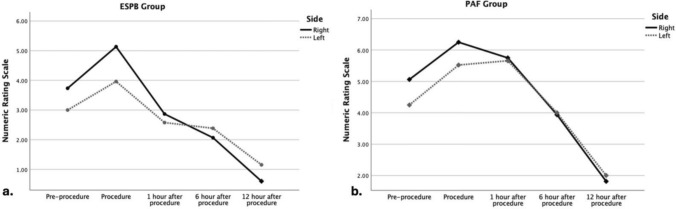
Median Numeric Rating Scale (NRS) scores according to laterality at each measument point for **a** erector spinae plane block (ESPB) or **b** procedural analgesia with fentanyl (PAF) groups

Regarding laterality, both ESPB and PAF groups were analyzed separately for right- and left-sided procedures, and the comparative results are presented in Fig. 3a, b. In both the ESPB and PAF groups, pre-procedural NRS scores were higher in patients undergoing right-sided procedures, (*p* = 0.062). A second significant difference was observed 12 h post-procedure, with left-sided procedures being associated with higher pain scores in both groups (*p* = 0.012).

Notably, no patients in the ESPB group experienced NRS scores ≥ 6 at any post-procedural time point, and no additional opioid analgesia was required. In contrast, in the PAF group, a significant proportion of patients experienced NRS scores ≥ 6, requiring postoperative tramadol administration according to standard pain management protocols.

## Discussion

This study assesses the analgesic efficacy of ESPB during PTBD compared to fentanyl-based standard procedural analgesia. PTBD is a preferred intervention for biliary obstructions when ERCP or surgery is unfeasible. The procedure induces both somatic and visceral pain due to trauma to soft tissues, liver capsule stretching, and bile leakage, which may affect procedural success and patient comfort [10]. These pain sensations are often perceived as referred pain in the epigastric and hypochondriac regions. The pain experienced by the patient during and after the procedure negatively affects both patient and operator comfort, potentially impacting procedural success and duration.

Enhanced Recovery After Surgery (ERAS) protocols favor multimodal analgesia to minimize opioid-related adverse effects, including respiratory depression, gastrointestinal dysfunction, nausea, and dependence, which can delay recovery and prolong hospitalization [11]. ESPB provides both somatic and visceral analgesia while reducing opioid requirements and associated complications [12–14].

Various analgesic techniques have been used during PTBD, including IV sedation, epidural, paravertebral, interpleural, and transversus abdominis plane (TAP) blocks, each with benefits and limitations [13, 15, 16]. Recently, hepatic hilar nerve block has also been proposed as an effective method for pain control, especially during procedures such as radiofrequency ablation of liver lesions [17]. Although its use in PTBD is not yet well established, it may represent a promising alternative for selected patients. While remifentanil and intrathecal morphine can be effective, they increase postoperative nausea and opioid use [18]. Epidural analgesia remains an established and highly effective technique for managing postoperative pain in major open abdominal surgery, particularly due to its well-documented benefits in visceral pain control, opioid-sparing effects, and enhanced recovery. However, its use should be carefully balanced against patient-specific risks and the objectives of enhanced recovery protocols, as factors such as hypotension and coagulopathy may limit its applicability in certain patient populations [14, 19–21]. Harshfield et al. found that epidural anesthesia provided effective intraoperative pain control in 91% of patients but carried a 1% hypotension risk, while IV sedation resulted in inadequate pain relief in 50% of cases [22].

Culp et al. demonstrated that fluoroscopy-guided paravertebral block (PVB) significantly reduced pain and opioid use during PTBD [23]. However, PVB remains technically challenging due to its proximity to deeper structures, which increases the risk of serious complications such as pneumothorax, vascular injury, and intrathecal spread [24, 25]. In contrast, ESPB is easier to administer and has a lower complication profile.

Interpleural block (IPB) has shown variable efficacy, with a 52% success rate and an 18% failure rate in PTBD patients [26]. Given its 2–6% pneumothorax risk, ESPB may offer a safer alternative.

The PROSPECT guidelines recommend TAP blocks, epidural analgesia, and NSAIDs for hepatobiliary interventions but do not provide specific recommendations regarding ESPB [19]. While our study did not record cumulative opioid doses, the absence of high NRS scores and lack of additional opioid requirements in the ESPB group suggest a potential benefit in this context. These preliminary findings may help inform future investigations into the role of ESPB in percutaneous biliary procedures.

In a case report by Bharati et al., ultrasound-guided oblique subcostal transversus abdominis plane (OSTAP) block was described as a modification of TAP block and an effective analgesic method during PTBD [27]. But the generalizability of this technique remains limited due to the lack of larger studies. Additionally, our study supports the potential role of ESPB in reducing opioid requirements during PTBD, which may reflect its contribution to visceral pain modulation alongside somatic analgesia [28, 29].

Studies on hepatobiliary pain management primarily focus on surgical patients [30]. While Mutlu et al. demonstrated ESPB’s efficacy in cholecystostomy, no prior research has evaluated its role in interventional radiology [9]. Considering the risks such as hypotension and pneumothorax reported in the literature with techniques like epidural, PVB, IPB, and TAP block, the absence of such complications in patients who received ESPB in our study suggests that ESPB may offer a safer profile.

Our study suggests that ESPB may contribute to improved pain control in the early post-procedural period compared to PAF, as reflected by lower pain scores at 1 and 6 h post-PTBD. While ESPB’s anatomical spread is thought to provide both somatic and potential visceral analgesia, the exact onset dynamicsof pain relief were not specifically evaluated in this study. This study has several limitations that should be considered. Its retrospective, single-center design limits the generalizability of the findings. ESPB was predominantly utilized in patients considered at higher risk, such as those with organ dysfunction or who were unsuitable for sedation, introducing potential selection bias. Pain assessment relied on subjective patient-reported NRS scores, which may be influenced by individual perception and peri-procedural factors, and was limited to five time points within the first 12 h, without long-term follow-up. Furthermore, the procedures were performed by a limited number of operators, potentially introducing operator-dependent variability.

Additionally, specific procedural parameters such as the number of puncture attempts required for biliary access and total procedure duration were not systematically analyzed. Therefore, potential differences in technical complexity between groups could not be fully assessed, representing another important limitation.

Moreover, subgroup analyses were not performed to evaluate the potential influence of patient-related factors such as gender, age, or general frailty on pain scores. These variables may have affected pain perception and outcomes, but were not specifically assessed in this study.

Future multicenter, randomized controlled trials are warranted to validate these findings and to further evaluate the role of ESPB in PTBD analgesia across broader patient populations and in combination with other techniques.

In conclusion, ESPB appears to provide superior postoperative pain control over fentanyl-based analgesia in PTBD with pain relief after procedure. Given its ease of administration and opioid-sparing benefits, ESPB represents a promising alternative, particularly for high-risk patients. Further randomized controlled trials are needed to refine its role in PTBD.

## Data Availability

The authors confirm that the data supporting the findings of this study are available within the article and its supplementary materials.
